# PARP-1 inhibitors sensitize HNSCC cells to APR-246 by inactivation of thioredoxin reductase 1 (TrxR1) and promotion of ROS accumulation

**DOI:** 10.18632/oncotarget.21277

**Published:** 2017-09-26

**Authors:** Zhi-Xian Yin, Wei Hang, Gang Liu, Yi-Shu Wang, Xiang-Feng Shen, Qian-Hui Sun, Dong-Dong Li, Yong-Ping Jian, Yang-He Zhang, Cheng-Shi Quan, Qinghua Zeng, Yu-Lin Li, Rui-Xun Zhao, Qiang Ding, Zhi-Xiang Xu

**Affiliations:** ^1^ Department of Otorhinolaryngology Head and Neck Surgery, Tianjin Huanhu Hospital, Tianjin, China; ^2^ Key Laboratory of Pathobiology, Ministry of Education, Norman Bethune College of Medicine, Jilin University, Changchun, China; ^3^ Division of Hematology and Oncology, Comprehensive Cancer Center, University of Alabama at Birmingham, Birmingham, Alabama, USA; ^4^ Department of Medicine, University of Alabama at Birmingham, Birmingham, Alabama, USA

**Keywords:** head and neck squamous cell carcinoma (HNSCC), PARP-1 inhibitors, p53 reactivators, reactive oxygen species (ROS), thioredoxin reductase 1 (TrxR1)

## Abstract

Head and neck squamous cell carcinoma (HNSCC) is the sixth most common cancer worldwide. Mutations of TP53 may reach 70% - 85% in HNSCC patients without human papillomavirus (HPV) infection. Recurrence rate remains particularly high for HNSCC patients with mutations in the TP53 gene although patients are responsive to surgery, irradiation, and chemotherapy early in the treatment. p53-Reactivation and Induction of Massive Apoptosis-1 (PRIMA-1) and its methylated analogue PRIMA-1^Met^ (also known as APR-246) are quinuclidine compounds that rescue the DNA-binding activity of mutant p53 (mut-p53) and restore the potential of wild-type p53. In the current report, we demonstrated that inhibition of poly (ADP-ribose) polymerase-1 (PARP-1) with 6(5H)-phenanthridinone (PHEN) and N-(6-Oxo-5,6-dihydrophenanthridin-2-yl)-(N, N-dimethylamino) acetamide hydrochloride (PJ34) sensitizes UMSCC1, UMSCC14, and UMSCC17A, three HNSCC cell lines to the treatment of APR-246. PHEN enhances APR-246-induced apoptosis, but not programmed necrosis or autophagic cell death in HNSCC cells. The PARP-1 inhibition-induced sensitization of HNSCC cells to APR-246 is independent of TP53 mutation. Instead, PARP-1 inhibition promotes APR-246-facilitated inactivation of thioredoxin reductase 1 (TrxR1), leading to ROS accumulation and DNA damage. Overexpression of TrxR1 or application of antioxidant N-acetyl-L-cysteine (NAC) depletes the ROS increase, reduces DNA damage, and decreases cell death triggered by APR-246/PHEN in HNSCC cells. Thus, we have characterized a new function of PARP-1 inhibitor in HNSCC cells by inactivation of TrxR1 and elevation of ROS and provide a novel therapeutic strategy for HNSCC by the combination of PARP-1 inhibitors and APR-246.

## INTRODUCTION

Head and neck squamous cell carcinoma (HNSCC) is the major type of head and neck cancer and ranks as one of the most common cancers worldwide. There are more than 700,000 new HNSCC cases diagnosed each year globally, with more than 350,000 deaths from the disease annually [1]. Despite the latest innovations in basic science and the improvement in clinical therapeutics, the overall 5-year survival rate for HNSCC remains low [2, 3]. Development of new therapeutic drugs or exploration of combination for conventional therapy is urgently needed to effectively treat HNSCC.

Mutations of the TP53 tumor suppressor gene are the most frequent of all somatic genomic alterations in HNSCC. It is reported that mutations of TP53 may reach 70% - 85% in HNSCC patients without human papillomavirus (HPV) infection [4–11]. Mutations of p53 lead to the loss of wild-type p53 function, the dominant-negative effect on the remaining wild-type p53, and even gaining oncogenic functions to promote tumorigenesis and progression [12–15]. Recurrence rate remains particularly high for HNSCC patients with mutations in the TP53 gene although patients are responsive to surgery, irradiation, and chemotherapy early in the treatment. These phenotypes render a potentially rationale for targeting TP53 mutations as a therapeutic intervention in HNSCC patients [16–23].

p53-Reactivation and Induction of Massive Apoptosis-1 (PRIMA-1) and its methylated analogue PRIMA-1^Met^ (also known as APR-246) are quinuclidine compounds that rescue the DNA-binding activity of mutant p53 (mut-p53) and restore the potential of wild-type p53 [16, 21, 24]. PRIMA-1 and APR-246 are converted to methylene quinuclidinone (MQ), a Michael acceptor that can bind covalently to cysteines in mutant p53 and unfold wild type p53, hence restoring the activity of p53 [20, 21, 25]. Treatment with PRIMA-1 or APR246 up-regulates p53 target genes such as BAX, PUMA and NOXA and activates caspases -2, -3 and -9 [26]. Studies have revealed that MQ may also induce cell death independently of p53 in different tumor types [26]. It was found that MQ modifies and converts antioxidant thioredoxin reductase 1 (TrxR1) into pro-oxidant NADPH oxidase, leading to ROS production [27–29]. APR-246 may also reduces glutathione (GSH) content, hence further elevating the levels of intracellular ROS and eventually leading to DNA damage induced by the oxidative stress [30].

PARP-1 is the most abundant and active enzyme in the PARP family. PARP-1 binds to both single- and double-stranded DNA breaks, although its role in SSB repair via the BER pathway has been most clearly defined [31]. Inhibition of PARP leads to the accumulation of SSBs that become DSBs at replication forks. If the DSB repair mechanisms are impaired or otherwise insufficient, cells are unable to repair DNA damage. PARP inhibition in these cells will lead to a high degree of genomic instability and eventually cell death [32]. Thus, PARP inhibitors are actively investigated and show considerable promise as sensitizers for DNA damage agents and as therapeutic drugs for cancer patients bearing mutations in DNA repair genes or impaired DNA repair function [32–35]. In this study, we report a novel function of PARP inhibitor in the inactivation of TrxR1 and promoting APR-246-facilitated ROS accumulation and DNA damage, thereby enhancing APR-246-induced apoptosis in HNSCC cells.

## RESULTS

### Inhibition of PARP sensitizes HNSCC cells to APR-246

**Figure 1:**
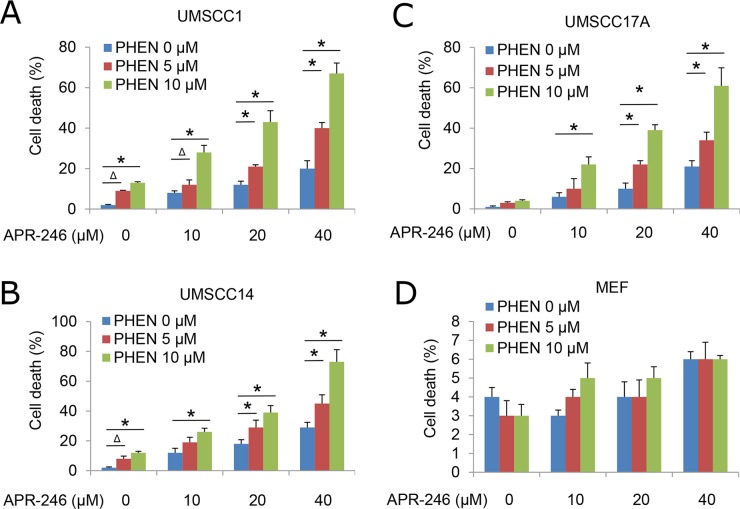
PARP Inhibitor PHEN sensitizes HNSCC cells to APR-246 Human HNSCC cell lines UMSCC1, UMSCC14, and UMSCC17A and primary MEFs were treated with different dosages of PHEN in the presence or absence of APR-246 for 72 h. Cell death was determined by the trypan blue exclusion assay. The assays were performed in triplicate samples, and the results are representative of three independent experiments (^Δ^ P < 0.05; ^*^ P < 0.01 as compared with PHEN 0 μM in each group). **(A)** UMSCC1; **(B)** UMSCC14 ; **(C)** UMSCC17A cells; and **(D)** MEFs.

To determine the impact of PARP inhibition on TP53 reactivator-induced HNSCC cell viability, we treated UMSCC1, UMSCC14, and UMSCC-17A with APR-246, 6(5H)-phenanthridinone (PHEN, a PARP-1 inhibitor), or their combination for 72 h. At dosages of 5 μM and 10 μM, PHEN alone only marginally induced cell death in HNSCC cells. Treatment with 10 - 40 μM APR-246 led to a modest reduction in the viability of HNSCC cells. Combination of PHEN with APR-246 resulted in a markedly enhanced overall cell death in a dose dependent manner and strikingly reduced IC50s (Figure 1A-1C, Supplementary Table 1). To assess the selectivity of the treatments to cancer (HNSCC) cells, we exposed primary MEFs to PHEN and/or APR-246. No noticeable cytotoxicity was observed in MEFs with the treatments at the dosages we detected (Figure 1D, Supplementary Table 1). Treatment with another PARP inhibitor, PJ-34, we obtained similar results in cell death after the treatment with or without APR-246 (Supplementary Figure 1).

### Inhibition of PARP-1 enhances APR-246-induced apoptosis in HNSCC cells

**Figure 2:**
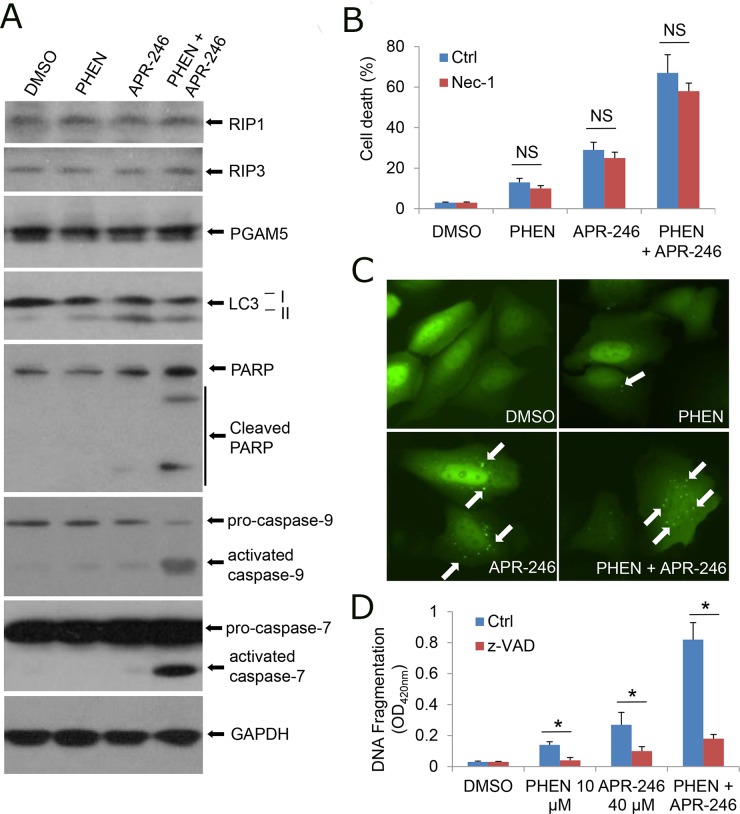
Inhibition of PARP-1 enhances APR-246-induced apoptosis in HNSCC cells **(A)** UMSCC14 cells were treated with 10 μM PHEN and/or 40 μM APR-246 for 24 h. After the treatment, whole cell extracts were collected for the western blot analysis. **(B)** UMSCC14 cells were treated with 10 μM PHEN and/or 40 μM APR-246 in the presence or absence of 20 μM necrostatin-1 (Nec-1) for 72 h. After the treatment, cell death was determined as described in Figure 1. n = 3, NS = non-significant. **(C)** UMSCC14 cells were transfected with pcDNA3/GFP-LC3 for 24 h. The cells were then treated with 10 μM PHEN and/or 40 μM APR-246 for additional 24 h. Autophagosomes/autolysosomes (GFP-LC punctuates) were monitored under an Olympus IX51 fluorescence microscope. White arrows show the autophagosomes/autolysosomes. **(D)** UMSCC14 cells were pretreated with 20 μM z-VAD-fmk for 6 h before addition of 10 μM PHEN and/or 40 μM APR-246 for 72 h. After the treatment, cell apoptosis was quantified using a cell death ELISA kit (Roche Diagnostics) showing enrichment of nucleosomes in the cytoplasmic fraction of the cells. Values represent the mean ± S.D. ^*^ P < 0.01; n = 3.

To characterize the cell death induced by PARP-1 and APR-246, we first determined whether the cell death is prompted by necrosis. Treatment with PHEN and/or APR-246 could not influence the expression of receptor interacting protein (RIP) kinases RIP1, RIP3, and phosphoglycerate mutase family member 5 (PGAM5), critical constituents triggering programmed necrosis (necroptosis) [36] (Figure 2A). PHEN and/or APR-246 associated cell death or cell viability was not affected by the pretreatment with necrostatin-1 (*Nec*-*1*), a selective RIP1 inhibitor [37] (Figure 2B), suggesting that PHEN and APR-246 at the current dosages (10 uM and 40 uM respectively) are unable to induce necroptosis. Autophagy was reported to be associated with non-apoptotic programmed cell death and PRIMA-1 was reported to be able to induce autophagy at a relatively high concentration [38]. We asked whether PHEN could enhance APR-246-induced cell death by promoting autophagy. Consistently, immunoblot assay showed that autophagy related gene 8 (ATG8), also called light chain 3 (LC3), underwent a conversion from the LC3-I isoform to the LC3-II isoform in APR-246-treated cells, indicating the induction of autophagy (Figure 2A). Autophagosome/autolysosome formation was observed in UMSCC14/GFP-LC3 cells treated with APR-246 (Figure 2C). However, PHEN could neither induce autophagy alone nor enhance APR-246-induced autophagy (Figure 2A & 2C), suggesting that PHEN-promoted cell death in APR-246-treated cells is not caused by autophagy.

PRIMA-1 and APR-246 were reported to induce apoptosis *in vitro* and *in vivo* [24–30]. To determine whether PHEN could enhance APR-246-induced cell death by promoting apoptosis, we detected apoptotic markers in the cell lysates. As shown in Figure 2A, the cleavage of PARP-1, caspase-9, and caspase-7 was markedly enhanced by the cotreatment with PHEN and APR-246. Detection of the cleaved DNA/histone complexes (nucleosomes) in the cells demonstrated the enrichment of nucleosomes in the cytoplasmic fraction of the cells co-treated with PHEN and APR-246, supporting the notion that the cell death is apoptosis (Figure 2D). To further confirm the induction of apoptosis by the cotreatment of PHEN and APR-246, cells were pretreated with benzyloxycarbonylvalyl-alanyl–aspartic acid (O-methyl)–fluoro-methylketone (zVAD-fmk), a pan-apoptotic inhibitor. As expected, the enrichment of nucleosomes in the cytoplasmic fraction of the cells co-treated with PHEN and APR-246 in the presence of zVAD-fmk was strikingly reduced although a small fraction of the cells still underwent cell death (Figure 2D), which may be due to additional non-apoptotic cell death. Taken together, we conclude that inhibition of PARP-1 enhances APR-246-induced apoptosis in HNSCC cells.

### PARP-1 inhibition-induced sensitization of HNSCC cells to APR-246 is independent of TP53 mutation

**Figure 3:**
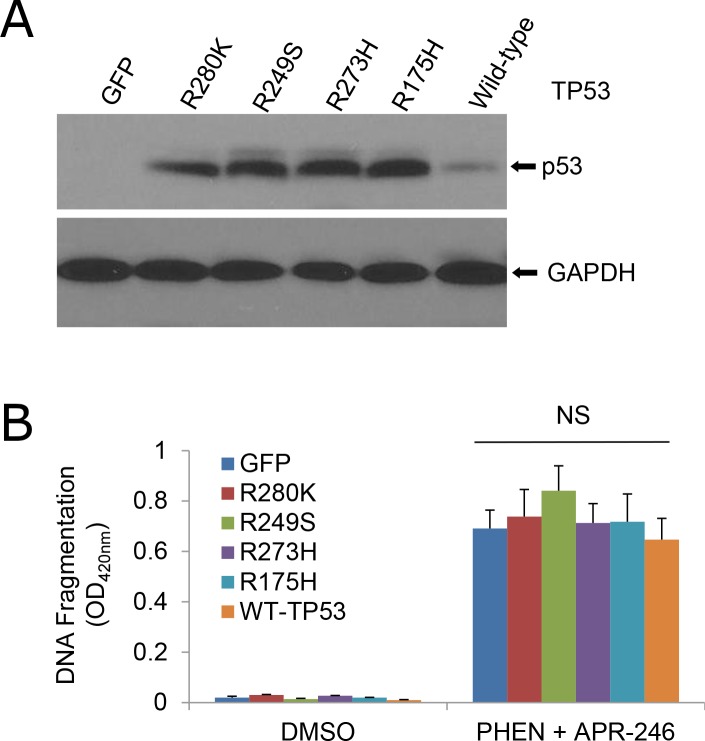
Sensitivity of cells to the cotreatment of PHEN and APR-246 is independent of TP53 mutation UMSCC1 cells were infected with lentiviruses expressing TP53 mutants R280K, R249S, R273H, and R175H, wild-type TP53, or GFP (control). Cell transduction efficiency was at least 60% with the fluorescence microscopy analysis at 48 h after the infection. **(A)** Immunoblot analysis of p53 in the transduced cells. **(B)** Apoptosis in the cells treated with 10 μM PHEN and 40 μM APR-246 for additional 72 h. Cell apoptosis was quantified using a cell death ELISA kit (Roche Diagnostics) showing enrichment of nucleosomes in the cytoplasmic fraction of the cells. The data represent the mean ± S.D. NS: Non-significant. n = 3.

PRIMA-1 and APR-246 were initially screened and developed as re-activators of the mutant p53 gene [20, 25]. Recent studies showed that the compounds may possess a broad function in addition to the suppression of mutant p53 and reactivation of the p53 functions [28–30]. To determine whether the cell death from the cotreatment of PHEN and APR-246 is dependent of p53 mutation, we compared cell viability in UMSCC1 (p53 deficient), UMSCC14 (p53 mutation), and UMSCC17A (wild-type p53) under the treatment of both agents. As shown in Figure 1 and Supplemtary Figure 1, all the three cell lines responded to the cotreatment although p53 mutation UMSCC14 cells seemed to be more sensitive to the treatment. To further confirm the observation, we transduced wild-type and mutation p53 constructs to UMSCC1 cells (Figure 3A). Consistently, cells with wild-type and mutant p53 showed a similar response to the co-treatment (Figure 3B). Taken together, our results suggest that PARP inhibition-induced sensitization of HNSCC cells to APR-246 is independent of TP53 expression status.

### PARP-1 inhibitor promotes ROS accumulation in HNSCC cells

**Figure 4:**
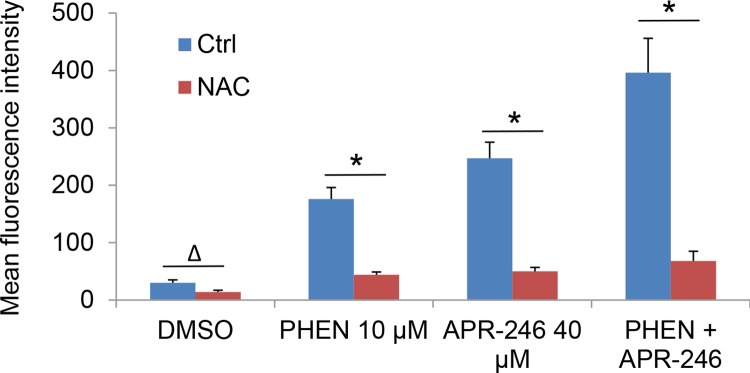
PARP-1 inhibition promotes the accumulation of ROS in APR-246-treated HNSCC cells, which is abrogated by pretreatment of antioxidant NAC UMSCC14 cells were treated with 10 μM PHEN and/or 40 μM APR-246 in the presence or absence of 5 mM NAC for 24 h. One hour prior to the termination of the treatment, 100 ng/ml dihydroethidium was added to the medium. The cells were collected, washed and analyzed by flow cytometry with the red laser channel (FL-3) using a FACscan analyzer. The data represent the mean ± S.D. ^Δ^ P < 0.05, ^*^ P < 0.01, n = 3.

PRIMA-1 is converted to methylene quinuclidinone (MQ), a Michael acceptor that can bind covalently to cysteines in mutant p53 and unfolded wild type p53, hence restoring the activity of p53 [25]. Studies have revealed that MQ may also induce cell death independently of p53 in different tumor types [16]. One such mechanism is the induction of reactive oxygen species (ROS) by disturbing the cellular redox balance [27]. To determine whether PARP inhibition is able to promote ROS accumulation in APR-246 treated cells, we analyzed ROS levels in PHEN and/or APR-246 treated cells. Indeed, intracellular levels of ROS were increased in UMSCC14 cells exposed to APR-246. Treatment of PHEN also modestly increased the ROS level in the cells. Strikingly, co-treatment of PHEN and APR-246 led to a 3-fold increase in intracellular ROS (Figure 4). The antioxidant N-acetyl-L-cysteine (NAC) reduced ROS levels in PHEN and/or APR-246-treated cells (Figure 4). Together, our data support the notion that PHEN treatment enhances the ROS accumulation in cells exposed to APR-246.

### PARP-1 inhibitor augments APR-246-induced TrxR1 suppression

**Figure 5:**
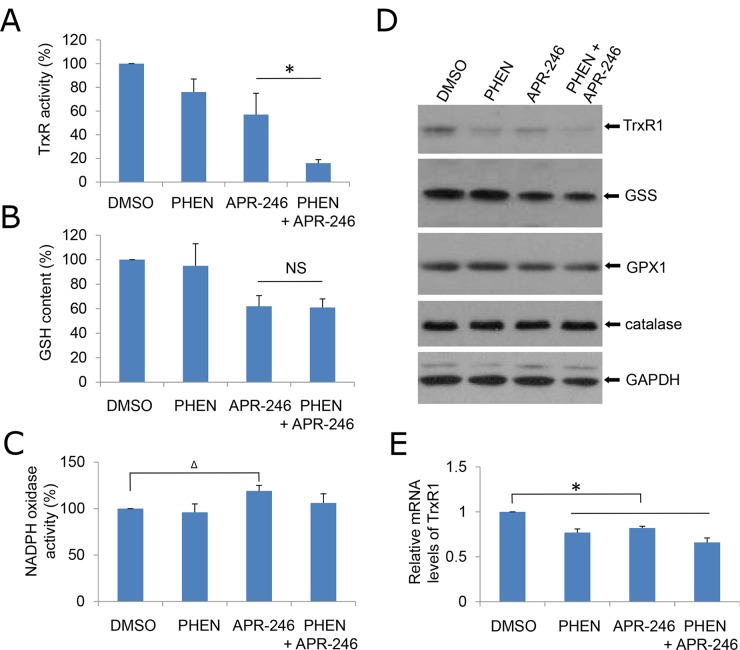
PHEN and ARP-246 suppress TrxR1 A total of 2 X 10^5^ cells were plated in six-well plates. The following day, cells were exposed to 10 μM PHEN and/or 40 μM APR-246 for 24 h. The cells were then lysed. The clarified supernatants were used for the analysis of TrxR enzymatic activity **(A)**, intracellular GSH concentration **(B)**, NADPH oxidase activity **(C)**, or immunoblot analysis **(D)** as described in Materials and Methods. **(E)** mRNA levels of TrxR1 in cells were determined by qPCR. The values in (A), (B), (C), and (E) were normalized to mg protein. The number in DMSO-treated cells was set as “100%” or “1”. The data represent means ± SD for 3 independent experiments. ^Δ^ P < 0.05, ^*^P < 0.01, NS: Non-significant.

Thioredoxin reductase 1 (TrxR1) is a cytoplasmic pyridine nucleotide oxidoreductase, which reduces thioredoxins (Trx) as well as other substrates. Trx executes antioxidant roles directly by reducing protein disulfides, as well as indirectly by donating reducing equivalents to peroxide scavenging enzymes such as peroxiredoxins or reductive repair enzymes such as methionine sulfoxide reductases or sulfiredoxin [39]. Peng et al. showed that APR-246/MQ is able to modify TrxR1 (antioxidant) and convert it to a dedicated NADPH oxidase (pro-oxidant), which can induce ROS production [27]. In addition, APR-246 can deplete glutathione (GSH) content, thus further increasing intracellular ROS levels and ultimately leading to DNA damage induced by the oxidative stress [30]. To determine how PHEN exposure promotes the ROS accumulation in APR-246-treated cells, we determine TrxR1, NADPH oxidase, and GSH activity in the cells. Consistent with previous report, treatment with APR-246 reduced the activity of GSH and TrxR1 and modestly increased NADPH oxidase activity (Figure 5A-5C). Exposure to PHEN could not further manipulate GSH and NADPH oxidase activity (Figure 5A & 5B), indicating that PHEN-promoted accumulation of ROS in APR-246-treated cells may not be attributable to the dysregulation of GSH content and NADPH oxidase activity. PHEN alone or combined with APR-246 markedly reduced the activity of TrxR1 in UMSCC14 cells (Figure 5A). Interestingly, treatments of PHEN and/or APR-246 also moderately reduced the expression of TrxR1 (Figure 5D & 5E), suggesting that the decreased TrxR1 activity upon the treatments in UMSCC14 cells may be due to both enzyme inhibition and decreased TrxR1 protein levels.

### TrxR1 is necessary for PARP-1 inhibitor - and APR-246 -induced ROS accumulation

**Figure 6:**
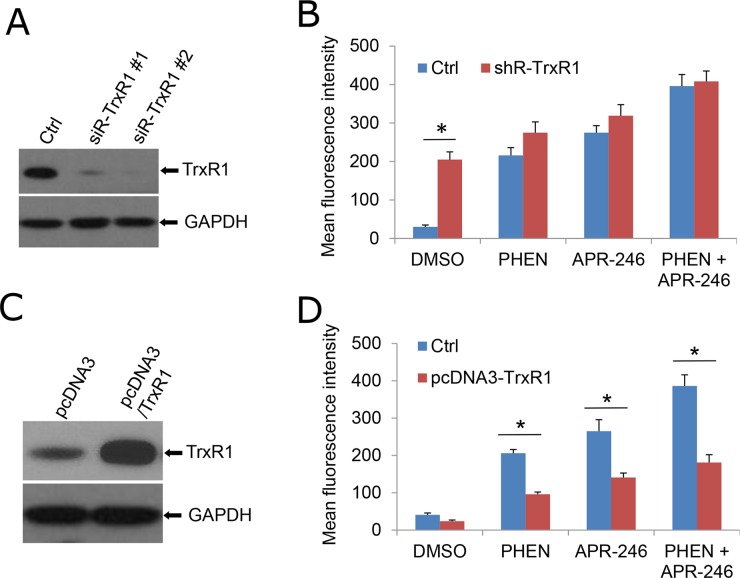
TrxR1 is necessary for PHEN- and APR-246-induced ROS accumulation **(A, B)** Knockdown of TrxR1 increases intracellular ROS levels. Two duplexes of TrxR1 siRNA were transfected into UMSCC14 cells. Forty eight hours after the transfection, the cells were collected for the confirmation of knock-down by western blot (A) or exposed to 10 μM PHEN and/or 40 μM APR-246 for additional 24 h (B). Cells were then collected for the intracellular ROS determination. Measurements of ROS were performed as described in Figure 4. **(C, D)** Over-expression of TrxR1 reduces PHEN and APR-246 induced increase of ROS. TXNRD1 (TrxR1) gene was amplified with RT-PCR and cloned into pcDNA3 vector. pcDNA3/TrxR1 was transfected into UMSCC14 cells. Forty eight hours after the transfection, the cells were collected for the determination of TrxR1 expression by western blot (C) or exposed to 10 μM PHEN and/or 40 μM APR-246 for additional 24 h (D). Cells were then collected for the intracellular ROS determination. Measurements of ROS were performed as described in Figure 4. ^*^ P < 0.01, n = 3.

To validate the role of TrxR1 in PHEN and APR-246-induced ROS accumulation, we knocked down TrxR1 in UMSCC14 cells followed by the cotreatment of PHEN and APR-246. It showed that PHEN could no longer further elevate ROS level in the presence or absence of APR-246 on the basis of an elevated ROS content as TrxR1 activity suppressed in the TrxR1-depleted cells (Figure 6A & 6B, Supplementary Figure 2A). In contrast, NADPH oxidase activity was only marginally reduced in cells with or without the cotreatment (Supplementary Figure 2B). Overexpression of TrxR1 prevented the cells from PHEN-induced elevation of ROS in PHEN treatment alone or combination with APR-246 (Figure 6C & 6D). Collectively, these results suggest that TrxR1 plays a critical role in PARP-1 inhibition-promoted elevation of ROS in HNSCC cells.

### APR-246 treatment induces DNA damage, which is enhanced by PARP inhibition

**Figure 7:**
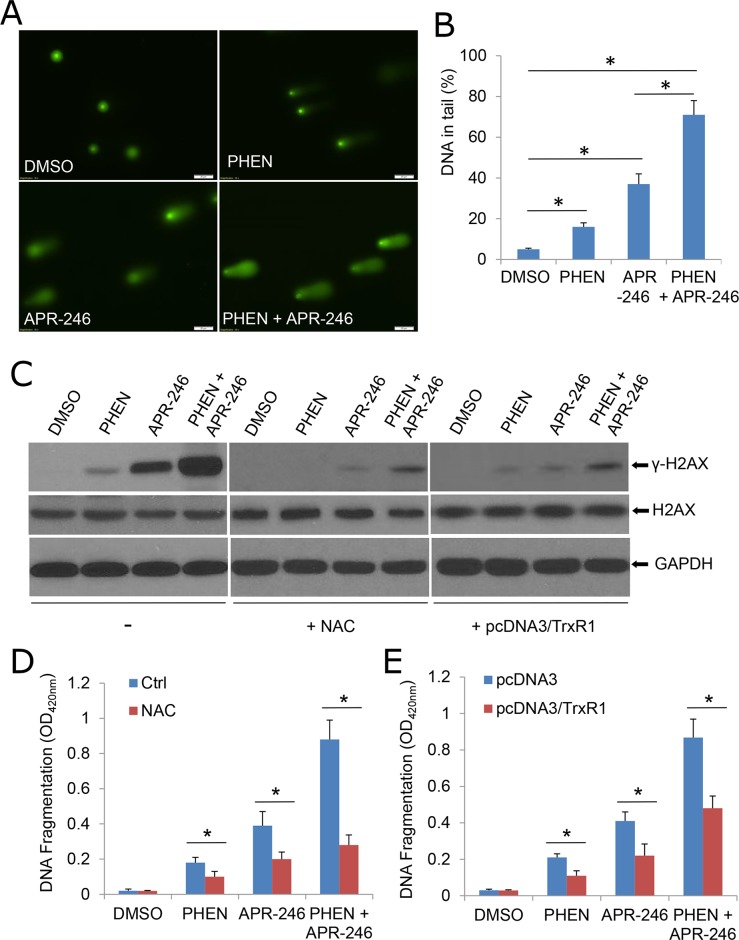
APR-246 treatment induces DNA damage, which is enhanced by PARP inhibition **(A, B)** Comet assay demonstrating elevated DNA damage in cells treated with PHEN and/or APR-246. UMSCC14 cells were treated with 10 μM PHEN and/or 40 μM APR-246 for 24 h. The cells were then trypsinized and washed with PBS. Two thousand cells were mixed with 100 μl low melting agarose for alkaline comet assay. Cells in the gel were stained and visualized with epifluorescence microscopy (A). (B) Percentage of DNAs in the tail (damaged DNA) was calculated. ^*^ P < 0.01, n = 3. **(C)** DNA damages were markedly escalated in PHEN- and APR-246-treated cells. Antioxidant NAC and over-expression of TrxR1 reduce above agents-induced DNA damage. The indicated UMSCC14 cells were exposed to 10 μM PHEN and/or 40 μM APR-246 for 24 h. Cell lysates were collected for western blot analysis. GAPDH serves as loading control. **(D, E)** NAC pretreatment and over-expression of TrxR1 reduce PHEN- and APR-246- induced apoptosis. UMSCC14 cells were pretreated with 5 mM NAC for 6 h (D) or transfected with pcDNA3/TrxR1 as described in Figure 6C and 6D (E). The cells were then exposed to 10 μM PHEN and/or 40 μM APR-246 for 48 h. After the treatment, cell apoptosis was quantified using a cell death ELISA kit (Roche Diagnostics) showing enrichment of nucleosomes in the cytoplasmic fraction of the cells. Data represent the mean ± S.D. ^*^ P < 0.01, n = 3.

PARP family proteins (mainly PARP-1 and PARP-2) participate in a physiological response against DNA damage and repair of single-strand DNA break (SSB)-induced DNA damage [31]. Lack of PARP activity with genetic modification or inhibitors increases SSB count. These unrepaired SSBs are converted into double-strand DNA breaks (DSBs) at replication fork [31]. If the DSB repair mechanisms are impaired or otherwise insufficient, cells are unable to repair DNA damage, which may lead to a high degree of genomic instability and cell death. To determine whether APR-246-initiated ROS accumulation leads to DNA damage, and more importantly, facilitated by cotreatment with PHEN, hence resulting in cell death, we exposed UMSCC14 cells to APR-246 and/or PHEN and measured the DNA damage by the comet assay. The tail moment that reflects the frequency of breaks was used to quantify DNA damage [40]. In APR-246- or PHEN-treated cells, the level of damage was considerably higher than that in DMSO-treated (control) cells (Figure 7A & 7B). Co-treatment with the two agents led to an obviously extended damaged DNA tail moment in cells (Figure 7A & 7B). γ-H2AX, another DNA damage marker, was also increased in APR-246 or PHEN treated cells and enhanced by the co-treatment (Figure 7C). In keeping with the rescuing role of NAC treatment (Figure 4) or TrxR1 transduction (Figure 6D) against ROS accumulation, pretreatment with NAC or over-expression of TrxR1 reduced DNA damage (Figure 7C) and cell apoptosis triggered by APR-246/PHEN (Figure 7D & 7E). Taken together, our results suggest that PHEN not only promotes the accumulation of ROS, but also facilitates DNA damage and cell death in APR-246-treated HNSCC cells.

## DISCUSSION

Cisplatin-containing chemotherapy or its combination with radiation has become a standard care for patients with locally advanced HNSCC [41]. However, the recurrence rate of the disease is still high and the long-term survival in patients with advanced-stage head and neck cancer remains poor. Recent genomic data have revealed that up to 85% of non-HPV infection-induced HNSCC patients bear TP53 mutation, making it the most frequently mutated gene in HNSCC [4–10]. TP53 is a tumor suppressor, which plays a critical role in cell cycle arrest, senescence, activation of checkpoints after DNA damage and genotoxic stresses. Mutations of p53 may not only lead to the loss of wild-type p53 functions but also cause the dominant-negative effect on the remaining wild-type p53 and even gain oncogenic functions to promote tumorigenesis and progression [12]. TP53 mutation is associated with poor therapeutic response and decreased survival in HNSCC [13–15, 42]. Thus, an important objective in the clinic is to develop therapeutic strategies for overcoming chemotherapy resistance in patients with TP53 mutations.

With compound screening assays, a couple of agents have been developed in targeting TP53 mutations and/or restoring wild-type TP53 activities [22, 43, 44]. For example, PRIMA-1, APR-246, MIRA-1, CP-31398, and ellipticine restore p53 function on transcriptional transactivation and induce cell death preferentially in TP53 mutation carrying tumors [22]. RITA, nutlins, and HLI98 restore the tumor suppressive function of p53 by inhibiting MDM2-mediated p53 degradation in wild-type TP53-bearing tumors. Pharmacologic restoration of the p53 pathway induces cell-cycle arrest and massive apoptosis of tumors without causing adverse effects on normal cells. Thus, reconstitution of the p53 pathway is becoming one of the most exciting novel therapeutic strategies against cancer. In addition, the crucial role of p53 in DNA damage response makes it an ideal target for combination strategies between p53 activators and DNA-damaging agents or compounds interacting with DNA damage repair pathways [29].

PRIMA-1 and its analogue APR-246 have been reported to rescue the DNA-binding activity of mutant p53 (mut-p53) and restore the potential of wild-type p53. Several studies have shown the relevance of combining APR-246 with chemotherapeutic agents, resulting in a synergistic effect and inhibition of chemotherapy resistance [22, 26, 29]. In addition, recent reports showed that PRIMA-1 and its analogue APR-246 may also bear functions on TP53 negative cells. PRIMA-1 and APR-246 are converted to methylene quinuclidinone (MQ), a Michael acceptor that covalently binds to cysteines in mutant p53 or unfolded wild type p53, thereby restoring its wild-type conformation [14, 21]. It was reported that MQ modifies TrxR1 and converts this enzyme from a reductase to a NADPH oxidase that promotes ROS production, which may eventually contribute to PRIMA-1/APR-246-induced cell death and probably explains the observed effects of PRIMA-1/APR-246 on p53 null cancer cells [27]. Hypoxia is a causative factor for high ROS in tumor cells. It was found that hypoxia sensitized SKBR3 breast cancer cells carrying mutant p53 to PRIMA-1 [42]. The combination of PRIMA-1 with peroxidase increased apoptosis and induction of Puma and Mn-SOD in MCF-7 breast carcinoma cells overexpressing mutant p53 [45]. These results indicate that the antitumor activity of PRIMA-1 is influenced by hypoxia and suggest that PRIMA-1 may be useful for addressing chemoresistance in hypoxic tumors. In the current study, we found that APR-246 is cytotoxic not only to TP53 mutated UMSCC14 cells but to TP53 deficient UMSCC1 and TP53 wild-type UMSCC17A as well (Figure 1). Further validation revealed that APR-246 inactivated TrxR1 and promoted the accumulation of ROS (Figures 5 and 6). Interestingly, APR-246 only modestly increased NADPH oxidase activity, which could not explain a marked effect of APR-246 on ROS accumulation in the cells. Suppression of TrxR1-mediated reduction of Trx and the resultant antioxidant activity depletion may also contribute to the elevation of ROS in APR-246 treated UMSCC14 cells.

We demonstrated that PARP-1 inhibitors, PHEN and PJ34, markedly enhanced APR-246-induced cell death in HNSCC cells (Figure 1 and Supplementary Figure 1). Previous reports showed that APR-246 treatment leads to apoptosis associated with cleavage of PARP, which is involved in DNA repair and programmed cell death [46]. Thus, PARP-1 inhibitors may promote PRIMA-1/APR-246 facilitated cell death through the enhanced DNA damage [29]. In addition, in the current study, we characterize a new mechanism by which PARP-1 inhibitor enhances the killing effect of APR-246 on HNSCC cells. We found that PARP-1 inhibitor, PHEN, suppressed the activity of TrxR1 and markedly increased the concentration of ROS in UMSCC14 cells (Figures 5 and 6). Overexpression of TrxR1 and application of antioxidant NAC significantly reduced ROS and cell death in HNSCC cells co-treated with APR-246 and PARP-1 inhibitor (Figures 4, 6C and 6D, 7D and 7E), further supporting our notion that TrxR1 is a target of PARP-1 inhibitor PHEN. It is unknown how PARP-1 inhibitor suppresses TrxR1. It appears that both the activity and the expression of TrxR1 are involved (Figure 5A, 5D, and 5E). PARP participates in several cellular functions in addition to DNA repair, such as inflammation, DNA methylation, chromatin modification and cell death [31]. PARP-1 regulates the chromatin structure, which inherently affects the accessibility of DNA. It has been demonstrated that PARP-1 can simultaneously bind multiple nucleosomes, which results in de-condensation of the compact chromatin structure that represses gene transcription [31]. Thus, PARP-1 inhibition may lead to the suppression of multiple transcriptional factors, such as NF-κB and Nrf2 (Nuclear factor erythroid-2-related factor 2). It was reported that TrxR1 and Nrf2 can reciprocally regulate each other [47–49]. Cadmium-induced TrxR1 gene expression is mediated by the activation of Nrf2 transcription factor and its binding to ARE in the TrxR1 gene promoter [49]. Thus, Nrf2 may be a potential node transducing the action of PARP-1 inhibitor to TrxR1.

Under physiologic conditions, normal cells maintain redox homeostasis by controlling the proper balance between ROS generation and elimination. The redox dynamics may fluctuate within a tolerable range. An increase of ROS may promote cell proliferation and survival, as in the case of many cancer cells. However, when the increase of ROS reaches a critical level (the threshold), it may overwhelm the cellular antioxidant capacity and trigger the cell-death process [50]. Cell death induced by many chemotherapeutic agents, histone deacetylase inhibitors, proteasome inhibitors, redox cycling agents, and PARP-1 inhibitors and APR-246 (this paper), all appear to increase oxidant stress in cells [50]. This common effect suggests that neoplastic cells may be more vulnerable to oxidant stress because they function with a heightened basal level of ROS-mediated signaling, which is required for the increased rate of growth [51]. Therefore, addition of an agent that increases ROS generation, or that decreases ROS scavenging capacity, may push the ROS level in the tumor to reach a threshold that may overwhelm the cellular antioxidant capacity and trigger the cell-death process [50]. Indeed, this concept was verified by the combined application of APR-246 and PARP-1 inhibitor (Figures 1 and 4). By contrast, normal cells exhibit a smaller increase in oxidant stress because their baseline levels of oxidant signaling are smaller, so the elevation of ROS or depletion of antioxidants presumably has less severe consequences for the cellular oxidative-redox environment [52]. Therefore, to the extent that ROS toxicity induced by certain chemotherapeutic agents or natural compounds can be an effective means of selectively eradicating malignant cells, it is useful to consider the most effective way to exploit this strategy in the future.

## MATERIALS AND METHODS

### Cell culture and drug treatment

Human HNSCC cell lines UMSCC1, UMSCC14, and UMSCC17 were originally from Thomas Carey at the University of Michigan and maintained in the lab [53]. Mouse embryonic fibroblasts were isolated from C57BL6 mice. Cells were cultured in DMEM supplemented with 10% fetal bovine serum (Atlanta Biologicals, Atlanta, GA) and maintained at 37°C under a humidified 5% CO_2_ atmosphere. Poly (ADP-ribose) polymerase (PARP) inhibitors 6(5H)-phenanthridinone (PHEN) and N-(6-Oxo-5,6-dihydrophenanthridin-2-yl)-(N, N-dimethylamino) acetamide hydrochloride (PJ34), and APR-246 were purchased from Sigma-Aldrich (St. Louis, MO). zVAD-fmk was purchased from Biomol International (Plymouth Meeting, PA).

### Plasmids, gene transfection, and establishment of over-expression stable cell lines

Wild-type and mutation p53 constructs (R280K, R249S, R273H, and R175H) were purchased from Addgene and sub-cloned into lentivirus vector [54]. UMSCC1 cells were transduced with lentiviruses expressing these mutations and selected with puromycin. Scramble siRNA and siRNA duplexes to human TrxR1 were ordered from Thermo Scientific (Lafayette, CO) and transfected to the cells with siRNA transfectant #2 (Thermo Scientific).

### Cell viability and cell death assay

Cell viability was measured by the MTT assay as described previously.^38^ Cell death was determined by trypan blue (Sigma-Aldrich, MO) exclusion assay. Cell apoptosis was quantified using a cell death ELISA kit (Roche Diagnostics) showing enrichment of nucleosomes in the cytoplasmic fraction of the cells.

### Fluorescence microscopy

UMSCC14 cells were grown in a six-well plate and transfected with pcDNA3/GFP-LC3 for 24 h. The cells were then treated with PHEN and/or APR-246. The autophagosomes/autolysosomes (GFP-LC3 punctuates) were monitored under an Olympus IX51 fluorescence microscope (Center Valley, PA, USA).

### Antibodies and western blot

After the indicated treatments, cells were lysed by ice-cold RIPA buffer containing proteasome-inhibitor cocktail (Cell Signaling, Danvers, MA). Protein samples were separated by SDS-PAGE, transferred onto a polyvinylidene difluride membrane (Thermo Scientific, Rockford, IL), and probed with an appropriate antibody. Antibodies against caspase-7, caspase-9, p53 were purchased from BD Pharmingen (San Diego, CA). Anti- human RIP1, RIP3, GPX1, Catalase, γ-H2AX, H2AX, and PGAM5 antibodies were from Abcam Inc. (Cambridge, MA). Anti-LC3 antibody was purchased from Novus Biological Inc. (Littleton, CO). TrxR1 antibody (B-2) and glutathione synthetase (GSS) antibody (C-5) were from Santa Cruz Biotechnology (Santa Cruz, CA). A total of 30 μg protein was used for the immunoblotting, unless otherwise indicated. GAPDH was used for the loading control.

### Detection of intracellular ROS

One hour prior to the termination of the treatment, 100 ng/ml dihydroethidium was added to the medium. The cells were harvested, washed, and analyzed by flow cytometry with the red laser channel (FL-3) using a FACscan analyzer [40]. ROS were also determined using a 96-well plate-based intracellular ROS assay as we previously reported [40].

### Measurement of enzyme activity of TrxR, NADPH oxidase, and GSH

A total of 2 × 10^5^ cells were plated in six-well plates. After 24 h-incubation, the cells were treated and lysed, and the clarified supernatants were used for the analysis of TrxR enzymatic activity, NADPH oxidase activity, intracellular GSH, and determination of total protein concentrations. Intracellular TrxR activity was measured using a Thioredoxin Reductase Assay Kit (Colorimetric) (ab83463) following the instructions of the manufacturer. The NADPH oxidase activity was meaured by the juglone-coupled assay as previously reported [27, 55]. A final concentration of 12.5 nM modified TrxR1, 200 mM NADPH and 50 mM juglone in TE buffer was used. The reaction was assessed by measuring NADPH consumption through the decrease of absorbance at 340 nm. Intracellular contents of GSH were determined using a GSH/GSSG Ratio Detection Assay Kit (Fluorometric-Green) (ab138881) according to the instructions of the manufacturer. Total protein concentrations were determined with a Bradford reagent kit (Bio-Rad Laboratories, Hercules, California).

### Real-time quantitative PCR

Total RNA was isolated with TRIZOL reagent (Life Technology, Grand Island, NY) and Direct-zol RNA miniPrep kit (Zymo Research, Irvine, CA). Comparative real-time polymerase chain reaction (qPCR) using SYBR Green SuperMix (Invitrogen, USA) was performed in a 96-well plate and was run in a 7500 Real-Time PCR System (Applied Biosystems) at 95°C for 10 minutes, followed by 40 cycles of 95°C for 15 seconds and 57°C for 1 minute. Each sample was analyzed in duplicate or triplicate.

### Alkaline comet assay

We followed the method for alkaline comet assay that we reported previously [40]. The cells were treated and run in alkaline buffer for 20 min, fixed, stained, and examined under the fluorescence microscope.

### Statistical analysis

Data were obtained from at least three independent experiments performed in triplicate and expressed as the means ± SD. Statistical significance was determined using the student’s *t*-test analysis, and p value < 0.05 was considered significant.

## SUPPLEMENTARY MATERIALS FIGURES AND TABLE


