# Supplementary material for: PARP-1 inhibitors sensitize HNSCC cells to APR-246 by inactivation of thioredoxin reductase 1 (TrxR1) and promotion of ROS accumulation
Source: Oncotarget. 2017 Sep 26;9(2):1885–97. doi: 10.18632/oncotarget.21277 (PMC5788606; doi:10.18632/oncotarget.21277)
Supplement: Supplementary file 1 [file oncotarget-09-1885-s001.pdf]

PARP-1 inhibitors sensitize HNSCC cells to APR-246 by inactivation of thioredoxin reductase 1 (TrxR1) and promotion of ROS accumulation

SUPPLEMENTARY MATERIALS

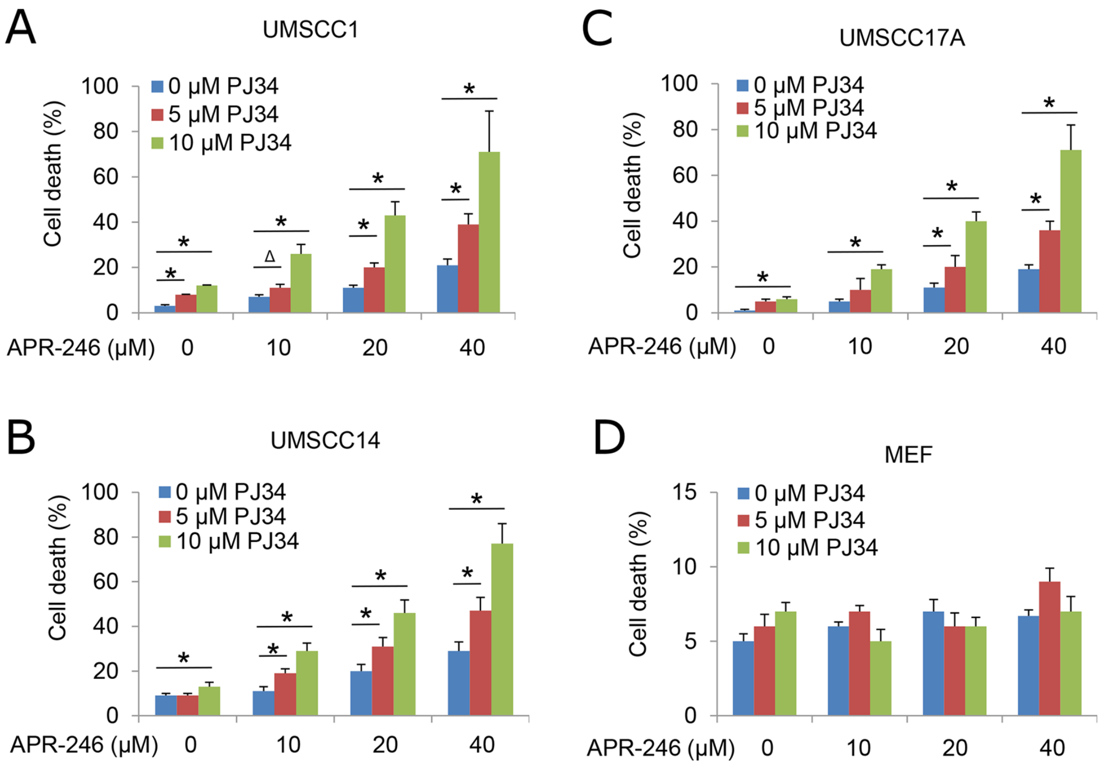

Supplementary Figure 1: PARP Inhibitor PJ34 sensitizes HNSCC cells to APR-246. Human HNSCC cell lines UMSCC1, UMSCC14, and UMSCC17A and primary MEFs were treated with different dosages of PJ34 in the presence or absence of APR-246 for 72 h. Cell death was analyzed as described in Figure 1. $^{\Delta}$ $P < 0.05$; * $P < 0.01$ as compared with 0 µM PJ34 in each group. **(A)** UMSCC1; **(B)** UMSCC14 ; **(C)** UMSCC17A cells; and **(D)** MEFs.

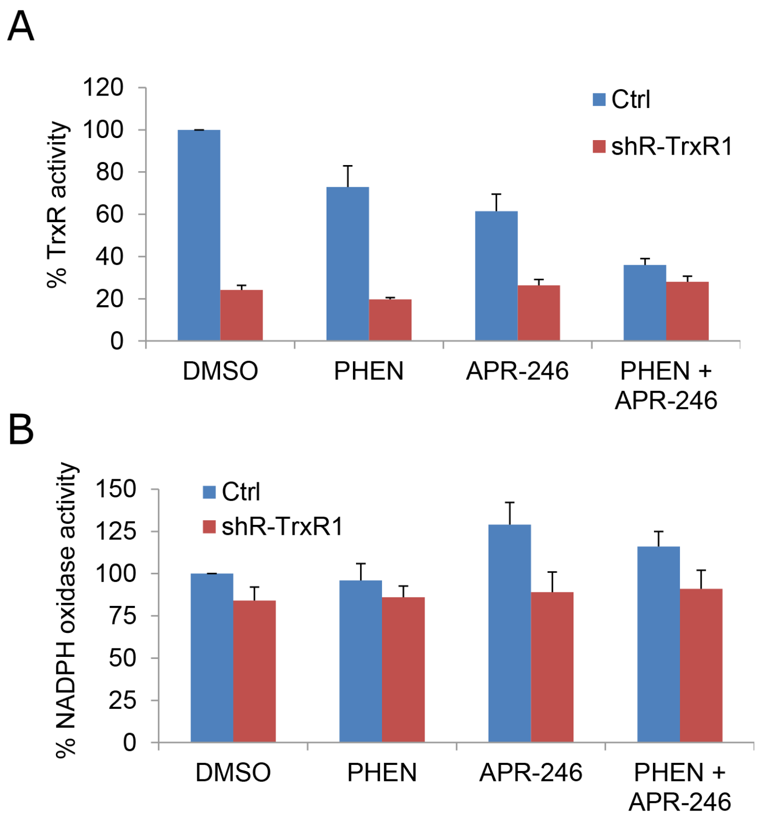

Supplementary Figure 2: TrxR1 and NADPH oxidase activity in TrxR1-knockdown cells treated with PHEN and/or APR-246. Knockdown of TrxR1 and treatment of PHEN and/or APR-246 in UMSCC14 cells were described in Figure 6. After the treatments, the cells were lysed and clarified supernatants were used for the analysis of TrxR **(A)** and NADPH oxidase activity **(B)**. The values were then normalized to mg protein. The number in DMSO-treated cells with shR-ctrl was set as "100%". The data represent means ± SD for 3 independent experiments.

Supplementary Table 1: IC50 for each treatment in Figure 1

Cells	PHEN	APR-246	PHEN + APR-246
UMSCC1	> 10 µM	> 40 µM	PHEN : 10 µM + APR-246: 24 µM<br>PHEN : 5 µM + APR-246: 48 µM
UMSCC14	> 10 µM	> 40 µM	PHEN : 10 µM + APR-246: 22 µM<br>PHEN : 5 µM + APR-246: 42 µM
UMSCC17A	> 10 µM	> 40 µM	PHEN : 10 µM + APR-246: 33 µM<br>PHEN : 5 µM + APR-246: 49 µM
MEF	> 10 µM	> 40 µM	PHEN >10 µM + APR-246 > 40 µM
